# Recurrence Beyond the Milan Criteria of HBV-Related Single Hepatocellular Carcinoma of 2–3 cm: Comparison of Resection and Ablation

**DOI:** 10.3389/fonc.2021.757149

**Published:** 2021-10-18

**Authors:** Shuanggang Chen, Weimei Ma, Lujun Shen, Ying Wu, Han Qi, Fei Cao, Tao Huang, Weijun Fan

**Affiliations:** ^1^ Department of Oncology, Yuebei People’s Hospital, Shantou University Medical College, Shaoguan, China; ^2^ Department of Minimally Invasive Interventional Therapy, Sun Yat-sen University Cancer Center, Guangzhou, China; ^3^ Department of Radiology, the Eighth Affiliated Hospital, Sun Yat-sen University, Shenzhen, China; ^4^ State Key Laboratory of Oncology in South China, Collaborative Innovation Center of Cancer Medicine, Sun Yat-sen University, Guangzhou, China

**Keywords:** hepatitis B virus, ablation techniques, surgical resection, recurrence beyond the Milan criteria, hepatocellular carcinoma

## Abstract

**Background:**

Comparison of patterns of early hepatocellular carcinoma (HCC) recurrence beyond the Milan criteria (HRBM) and identification of the independent risk factors of time to recurrence beyond the Milan criteria (TRBM) after resection or ablation can develop an optimal first-line treatment and provide more opportunities and waiting time for salvage transplantation.

**Methods:**

The patterns of HRBM after first-line resection or ablation in 384 patients with single-nodule HBV-associated HCC of 2–3 cm were retrospectively analyzed by one-to-one propensity score matching (PSM) between December 2008 and December 2017. The median TRBM between the resection group and the ablation group was estimated by Kaplan–Meier curves. The Cox regression analysis and binary logistic regression were used for the identification of the independent risk factors of TRBM and the occurrence of HRBM, respectively. The abilities of HRBM and the recurrence to predict overall survival (OS) were compared by the time-dependent receiver operating characteristic curves and estimated area under the curve.

**Results:**

Of 384 patients enrolled in our study, 260 (67.7%) received resection (resection group) and 124 (32.3%) underwent ablation (ablation group). The median TRBM in the resection group was significantly longer than that in the ablation group before PSM (median, not available *vs*. 101.4 months, *P* < 0.001) and after PSM (median, not available *vs*. 85.7 months, *P* < 0.001). Cox regression showed ablation, older age, CRP ≥1.81 mg/L, and PLT ≤80 × 10^9^/L were the independent risk factors of TRBM. Binary logistic regression also showed that ablation, CRP ≥1.81 mg/L, and PLT ≤80 × 10^9^/L were the independent risk factors of the occurrence of HRBM. The incidences of various phenotypes of HRBM were not significantly different between the two groups, but the incidence of HRBM at the first recurrence in the ablation group was significantly higher than that in the resection group (*P* < 0.05). Besides, compared with recurrence, HRBM was a better predictor of OS (*P* < 0.05).

**Conclusions:**

Compared with ablation, resection should be considered as a more appropriate first-line option for patients with single-nodule HBV-associated HCC of 2–3 cm and a more promising bridge for liver transplantation in those patients.

## Highlights

- HBV-associated HCC patients with a single nodule of 2–3 cm undergoing resection have significantly longer median TRBM and lower incidence of HRBM than those who received ablation, which suggested resection can provide them with more time and opportunities for salvage liver transplantation.
- In addition to treatment (ablation), serum CRP ≥1.81 mg/L and PLT ≤80 × 10^9^/L were the independent risk factors of TRBM and the occurrence of HRBM.
- The phenotype of tumor size and/or number (i.e., at least one within three lesions >3 cm, or one lesion >5 cm, or the number of lesions >3, and no evidence of extrahepatic metastasis and vascular invasion) was the most common pattern and accounted for more than 50% whether after radical resection or ablation or not.

## Introduction

Approximately 50%~80% of hepatocellular carcinoma (HCC) is associated with hepatitis B virus (HBV) infection worldwide (1). At present, ablation is regarded as first-line therapy for patients with early HCC <2 cm in addition to liver transplantation and resection (2, 3). However, whether ablation is suitable as a first-line option for patients with early HCC of 2–3 cm is controversial due to differences in the efficacy of resection and ablation in different randomized controlled trials, meta-analyses, and a real-world study (4–11), and we assume that these differences may be related to the differences in the patterns of HCC recurrence beyond the Milan criteria (HRBM) after curative ablation and resection, because some studies have shown that patients with HRBM have a worse prognosis (12, 13). Unfortunately, no study compares the differences in HRBM patterns after curative ablation and resection for HCC of 2–3 cm within the Milan criteria. Besides, a recent study have shown that compared with re-ablation or re-resection, salvage liver transplantation can significantly improve the survival rate of patients with the recurrent HCC within the Milan criteria (14). Those studies suggest that an optimal first-line option for patients with single-nodule HBV-associated HCC of 2–3 cm can be developed by comparison of the patterns of HRBM after resection and ablation and identification of independent risk factors of HRBM.

Therefore, the main purpose of this present study aims to compare the difference in the patterns of HRBM after resection and ablation for patients with single-nodule HBV-associated HCC of 2–3 cm within the Milan criteria as first-line options and identify the independent risk factors of HRBM after radical resection and ablation for those patients. The secondary purpose is to identify the independent risk factors of recurrence-free survival (RFS) and overall survival (OS) of those patients.

## Materials and Methods

### Patients

**Figure 1:**
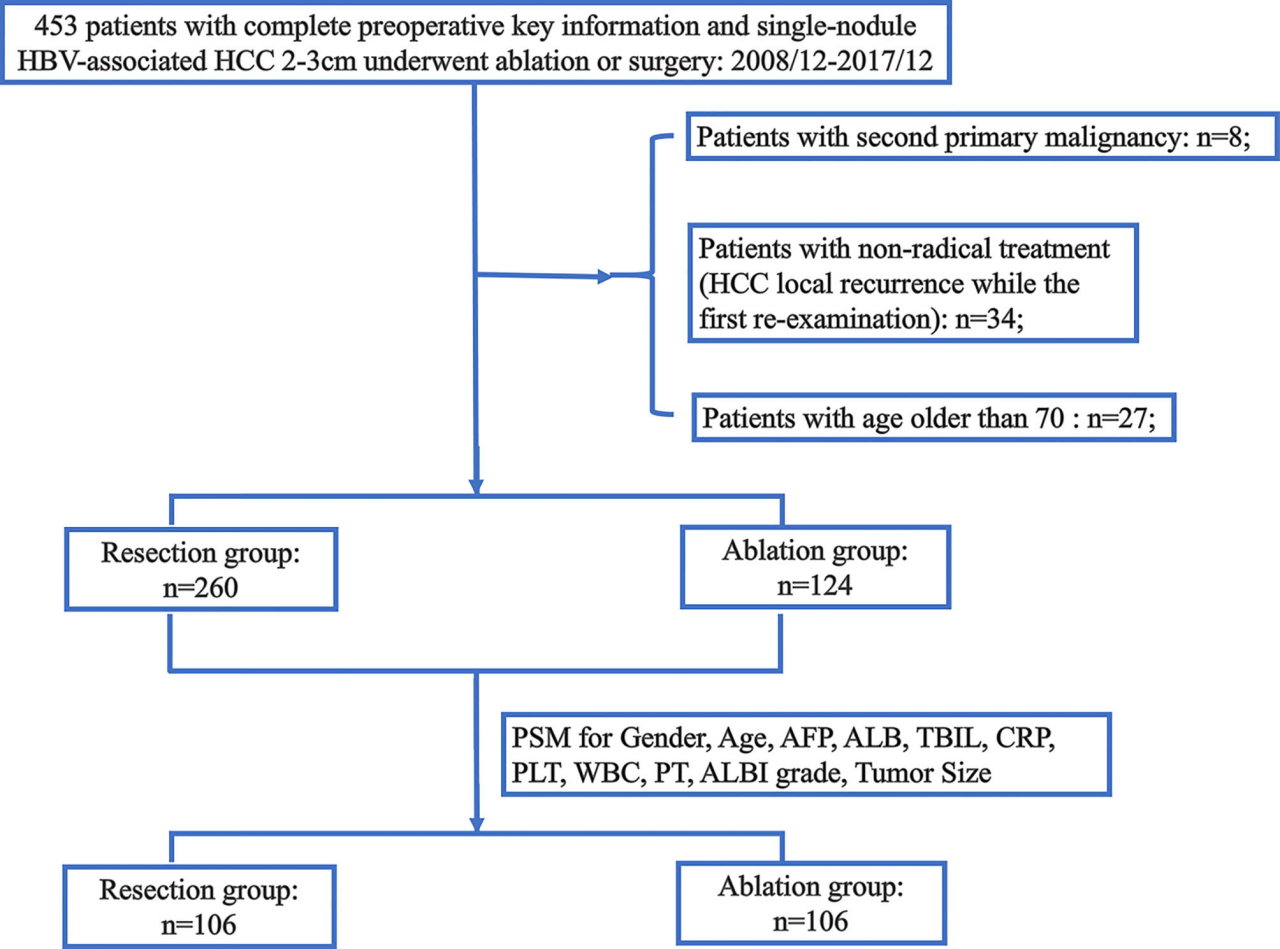
The flowchart of the selection. HCC, hepatocellular carcinoma; HBV, hepatitis B virus; AFP, alpha-fetoprotein; ALB, albumin; TBIL, total bilirubin; CRP, C-reactive protein; PLT, platelet; WBC, white blood cell; PT, prothrombin time; ALBI, albumin–bilirubin; PSM, propensity score matching.

We retrospectively analyzed a total of 453 HCC patients with single-nodule HCC of 2–3 cm who underwent surgical resection and ablation at the Sun Yat−sen University Cancer Center (SYSUCC) between December 2008 and December 2017. The inclusion criteria were as follows: 1) patients with HCC confirmed by pathological or imaging examination, 2) patients undergoing radical surgical resection and ablation as first-line treatment options (no HCC local recurrence while the first re−examination), 3) patients with hepatitis B virus infection and with single-nodule HCC of 2–3 cm, 4) patients with good liver function (albumin–bilirubin grade 1 or 2), 5) patients with the age of 18–70 years, 5) patients with no other treatments for HCC before recurrence, and 6) patients with no evidence of extrahepatic metastasis and vascular invasion. The exclusion criteria were as follows: 1) patients who are over 70 years old, 2) patients with non-radical treatment (HCC local recurrence while the first re−examination), and 3) patients with second primary malignancy. A total of 384 patients with single-nodule HBV-associated HCC of 2–3 cm were included, consisting of 260 (67.7%) patients who received surgical resection (resection group) and 124 (32.3%) patients who received ablation (ablation group). A flowchart is shown in **Figure 1**.

Informed consent was waived because of the retrospective nature of this study. This study was conducted in accordance with the Declaration of Helsinki and was approved by SYSUCC Hospital Ethics Committee.

### Treatment Protocols

#### Surgical Resection

Patients underwent anatomic partial hepatectomy performed with a negative resection margin in a standardized fashion. In short, intraoperative ultrasound is routinely performed to assess the relationship between tumor and blood vessels. A complete anatomic resection can be achieved by the resulting area of discoloration after ligation of the feeding vessels. The Pringle maneuver was routinely prepared for unexpected hemorrhage, with a switch of unclamp and clamp time of 5 and 10 min (15). All procedures were performed by experienced surgeons, who had at least 5 years of experience in hepatobiliary surgery.

#### Ablation

Ablation [microwave ablation (MWA) or radiofrequency ablation (RFA)] was performed by the radiologists with at least 3 years of experience in interventional therapy under computed tomography (CT) or real-time ultrasound (US). Both MWA and RFA were administered after anesthesia. These two operations usually went through four stages under aseptic conditions: puncture point positioning, puncture implementation, ablation implementation, and ablation endpoint evaluation. In order to eliminate tumor, the power and corresponding time were chosen according to location and size. Briefly, for tumor with a maximum diameter of 3 cm or less, a single ablation antenna was usually performed. The endpoint of ablation was defined as the security boundary extended at least 5–10 mm beyond the tumor boundary (16).

### Propensity Score Analysis

Propensity score matching (PSM) was used to minimize bias caused by differences of clinical characteristics associated with treatment selection and prognosis (17). Possible variables, including age, gender, serum alpha-fetoprotein (AFP), albumin (ALB) level, total bilirubin (TBIL) level, C-reactive protein (CRP) level, platelet (PLT) counts, white blood cell (WBC) counts, prothrombin time (PT), albumin–bilirubin (ALBI) grade, and tumor size, were comprehensively selected for 1:1 PSM analysis. Moreover, the matching tolerance of the propensity score was 0.06, which could evaluate the accuracy of matching between the two groups.

### Follow-Up and the Endpoint

Patients were re-examined using contrast agent-enhanced CT, US, or magnetic resonance imaging (MRI) about a month after treatment (ablation and resection). If there were no obvious signs of recurrence, follow-up about every 2–3 months within the first 2 years was conducted for those patients. Those patients were followed up once about 3–6 months from 2 to 5 years after treatment and then once about 6–12 months after 5 years until obvious signs of recurrence. The primary outcome, time to recurrence beyond the Milan criteria (TRBM), was defined as the interval time from initial treatment (ablation and resection) to the HRBM (12). The HRBM was defined as at least one within three lesions >3 cm, one lesion >5 cm, the number of lesions >3, extrahepatic metastasis, or vascular invasion at any time during the follow-up (12). The other interesting outcome is the identification of independent risk factors of overall survival (OS) and recurrence-free survival (RFS). The RFS was defined as the interval time from initial treatment to HCC recurrence or death caused by tumor. The OS was defined as the interval time from initial treatment (ablation and resection) to death by any cause. The median follow-up time was 48.13 (IQR 26.68–76.83) months in the entire cohort, 60.25 (IQR 29.52–78.98) months in the resection group, and 37.71 (IQR 23.49–68.74) months in the resection group, respectively.

### Statistical Analysis

Continuous variables without meeting the normal distribution were depicted by median and interquartile range (IQR). The mean and standard deviation were used for describing for normally distributed continuous variables. The comparison of continuous variables depended on the *t*-test and Mann–Whitney *U* test, and the comparison of categorical variables depended on the *χ* ^2^ test and the Fisher’s exact test. The median TRBM between the different groups was estimated by Kaplan–Meier curves, compared by the log-rank test and predicted by the univariate and multivariate Cox regression. The risk factors of the occurrence of HRBM were predicted by the binary logistic regression. The method of binary logistic regression is forward. The optimal cutoff values of CRP level (1.81 mg/L) and PLT counts (80 × 10^9^/L) for the Cox regression were based on our previous study that used survival ROC to find the ideal cutoff values (18). The time-dependent receiver operating characteristic (ROC) curves and the estimated area under the curve (AUC) were used for comparing the ability of HRBM and recurrence to predict overall survival (19). Analyses were two-sided, and *P <*0.05 indicated statistical significance. Statistical analyses were conducted using R version 3.6.1 (https://www.r-project.org/) and SPSS version 25.0 (IBM, United States).

## Results

### Study Population

The baseline characteristics of the patients with single-nodule HBV-associated HCC of 2–3 cm, who were treated with radical resection or ablation between December 2008 and December 2017, are displayed in **Table 1** before and after PSM. Before PSM, our study included a total of 384 HCC patients, of whom 260 (67.7%) and 124 (32.3%) were in the resection group and ablation group, respectively. After PSM, a total of 212 HCC patients were enrolled in our study, of which 106 (50%) patients were included in the resection group and the remaining 106 (50%) patients were included in the ablation group. Several variables, including ALB level (*P* = 0.009), TBIL level (*P* = 0.004), WBC counts (*P* = 0.001), PLT counts, age, PT, tumor size, and ALBI grade (*P* < 0.001), were statistically and significantly different between the resection group and ablation group before PSM; after PSM, the statistical significant differences were eradicated. There were no significant differences in other variables between the two groups before and after PSM (**Table 1**).

**Table 1:** Baseline characteristics of patients before and after propensity score matching.

	Before PSM			After PSM		
Characteristics	Resection*n* = 260 (%) or median (IQR)	Ablation*n* = 124 (%) or median (IQR)	*P*	Resection*n* = 106 (%) or median (IQR)	Ablation*n* = 106 (%) or median (IQR)	*P*
Gender			0.697			0.714
Male	220 (84.6)	103 (83.1)		87 (82.1)	89 (84.0)	
Female	40 (15.4)	21 (16.9)		19 (17.9)	17 (16.0)	
Age (years)	50 (15)	56 (14)	<0.001	53 (15)	55 (16)	0.357
AFP (ng/ml)	58.4 (463.2)	52.4 (369.7)	0.606	82.2 (418.4)	47.6 (446.2)	0.530
ALB (g/L)	43.1 (5.0)	41.9 (6.8)	0.009	42.1 (5.3)	42.2 (6.3)	0.394
TBIL (µmol/L)	13.5 (6.7)	15.3 (10.9)	0.004	14.0 (7.8)	14.6 (10.7)	0.176
CRP (mg/L)	1.21 (2.07)	1.42 (1.64)	0.947	1.14 (2.25)	1.46 (1.57)	0.735
PLT (×10^9^/L)	163.0 (82.0)	130.8 (90.8)	<0.001	136.0 (80.7)	143.5 (86.0)	0.786
WBC (×10^9^/L)	5.96 (2.01)	5.34 (2.18)	0.001	5.75 (1.92)	5.68 (2.22)	0.797
PT (s)	11.9 (1.20)	12.4 (1.70)	<0.001	12.1 (1.25)	12.2 (1.45)	0.099
Size (mm)	26 (5)	24 (5)	<0.001	24.5 (5)	24.0 (5)	0.978
ALBI			<0.001			0.646
Grade 1	218 (83.8)	82 (66.1)		78 (73.6)	75 (70.8)	
Grade 2	42 (16.2)	42 (33.9)		28 (26.4)	31 (29.2)	

Values in parentheses are percentages. Parameter data were compared by the Mann–Whitney U test. Categorical data were compared by using the test. Significance level: 0.05.

AFP, alpha-fetoprotein; ALB, albumin; TBIL, total bilirubin; CRP, C-reactive protein; PLT, platelet; WBC, white blood cell; PT, prothrombin time; ALBI, albumin–bilirubin.

### Time to Recurrence Beyond the Milan Criteria

**Figure 2:**
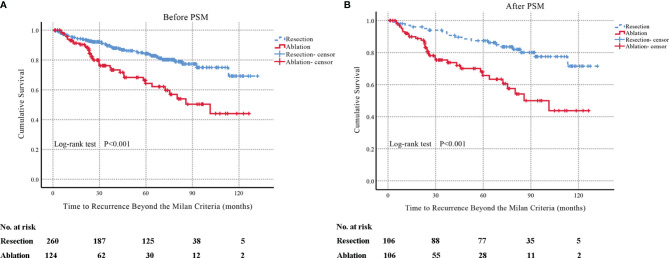
Before one-to-one PSM, Kaplan–Meier curves of TRBM in patients with single-nodule HBV-associated HCC of 2–3 cm who underwent resection or accepted ablation (resection group: *n* = 260; the median TRBM, not available; 95% CI: not available; ablation group: *n* = 124; the median TRBM, 101.4 months; 95% CI: 67.3–135.5 months; *P* < 0.001) **(A)**. After one-to-one PSM, Kaplan–Meier curves of TRBM in patients with single-nodule HBV-associated HCC of 2–3 cm who underwent resection or accepted ablation (resection group: *n* = 106; the median TRBM, not available; 95% CI: not available; ablation group: *n* = 106; the median TRBM, 85.7 months; 95% CI: 57.2–114.2 months; *P* < 0.001) **(B)**. TRBM, time to recurrence beyond the Milan criteria; PSM, propensity score matching; HBV, hepatitis B virus.

Before PSM, 20.6% (79/384) patients had a final HRBM during follow-up, of which 16.2% (42/260) patients were in the resection group and 29.8% (37/124) patients were in the ablation group (*P* = 0.002). The median TRBM was not available (95% CI: not available) in the resection group and 101.4 months (95% CI: 67.3–135.5 months) in the ablation group (*P* < 0.001) (shown in **Figure 2A**). The cumulative rates of HRBM in the resection group at 1, 3, 5, and 8 years were 4.4%, 10.4%, 15.7%, and 25.0%, respectively. The cumulative rates of HRBM in the ablation group at 1, 3, 5, and 8 years were 6.9%, 23.8%, 35.7%, and 49.7%, respectively.

After one-to-one PSM, 24.5% (52/212) patients had eventual HRBM during follow-up, of which 17.9% (19/106) patients were in the resection group and 31.1% (33/106) patients were in the ablation group (*P* = 0.025). The median TRBM was not available (95% CI: not available) in the resection group and 85.7 months (95% CI: 57.2–114.2 months) in the ablation group (*P* < 0.001) (shown in **Figure 2B**). The cumulative rates of recurrence exceeding the Milan criteria in the resection group at 1, 3, 5, and 8 years were 3.0%, 7.1%, 12.6%, and 22.5%, respectively. The cumulative rates of recurrence exceeding the Milan criteria in the ablation group at 1, 3, 5, and 8 years were 8.0%, 24.6%, 34.3%, and 50.0%, respectively.

### Analysis of Risk Factors of TRBM

All baseline variables in **Table 1** were included into time-dependent Cox regression for univariate analysis step by step, which showed that age, ALB level, CRP level, PLT counts, PT, ALBI grade, and treatment (ablation *vs*. resection) may be important predictors of TRBM. Moreover, these seven variables were incorporated into the time-dependent multivariate Cox regression showing that older age, serum CRP ≥1.81 mg/L, PLT ≤80 × 10^9^/L, and treatment (ablation) were the independent risk factors of TRBM (**Table 2**).

**Table 2:** Univariate and multivariate analyses of time to recurrence beyond the Milan criteria (TRBM) in the entire cohort.

Variable	Univariate analysis		Multivariate analysis	
	HR (95% CI)	*P*-value	HR (95% CI)	*P*-value
Gender (female *vs*. male)	0.980 (0.518–1.853)	0.949	–	–
Age (years)	1.029 (1.006–1.053)	0.013	1.028 (1.004–1.051)	0.021
AFP (ng/ml)	1.000 (1.000–1.000)	0.852	–	–
ALB (g/L)	0.938 (0.889–0.991)	0.021	–	0.770
TBIL (µmol/L)	1.008 (0.983–1.034)	0.532	–	–
CRP (≥1.81 *vs*. <1.81 mg/L)	1.570 (1.007–2.449)	0.046	1.755 (1.117–2.760)	0.015
PLT (≤80 × 10^9^/L *vs*. >80 × 10^9^/L)	3.331 (2.033–5.456)	<0.001	2.715 (1.641–4.492)	<0.001
WBC (×10^9^/L)	0.925 (0.807–1.059)	0.259	–	–
PT (s)	1.107 (0.999–1.228)	0.053	–	0.890
Size (mm)	1.003 (0.936–1.076)	0.928	–	–
ALBI (grade 2 *vs*. grade 1)	2.405 (1.516–3.816)	<0.001	–	0.317
Treatment (ablation *vs*. resection)	2.421 (1.552–3.775)	<0.001	2.266 (1.427–3.597)	0.001

Data in parentheses are 95% CIs. The data were analyzed using the Cox regression, and the variables with P-value <0.1 in univariate Cox regression were entered into the multivariate model. Significance level: 0.05.

HR, hazard ratio; AFP, alpha-fetoprotein; ALB, albumin; TBIL, total bilirubin; CRP, C-reactive protein; PLT, platelet; WBC, white blood cell; PT, prothrombin time; ALBI, albumin–bilirubin.

### Patterns of HCC Recurrence Beyond the Milan Criteria After Radical Resection and Ablation

The comparison of patterns of HRBM between radical surgical resection and ablation before PSM suggested that the incidences of various phenotypes of HRBM were not significantly different between the two groups (*P* > 0.05), but the incidence of HRBM in the ablation group was significantly higher than that in the resection group (*P* = 0.002), regardless of whether it is the first recurrence (*P* = 0.042) or other time periods (*P* = 0.036) during the follow-up (**Table 3**). These results were further clarified by PSM, but this result that the incidence of HRBM in the ablation group was significantly higher than that in the resection group (*P* = 0.025) was reflected in the first recurrence (*P* = 0.015), rather than other time (*P* = 0.549) (**Table 4**). In addition, it was worth noting that HRBM occurred in 41 (10.7%) patients at first recurrence despite close surveillance, of which 22/260 (8.5%) were in the resection group and 19/124 (15.3%) in the ablation group before PSM (*P* = 0.042) (**Table 3**), and this result was further demonstrated by PSM analysis (*P* = 0.025) (**Table 4**). Also, the binary logistic regression showed serum CRP ≥1.81 mg/L, PLT ≤80 × 10^9^/L, and treatment (ablation) were the independent risk factors for the occurrence of HRBM (**Table 5**).

**Table 3:** Patterns of HRBM after resection or ablation as first-line therapy for patients with single-nodule HBV-associated HCC of 2–3 cm before PSM.

Patterns of HRBM	Total*n* = 384 (%)	According to treatment		
		Resection (*n* = 260)	Ablation (*n* = 124)	*P*-value
Recurrence beyond the Milan criteria^a^	79 (20.6)	42 (16.2)	37 (29.8)	0.002
At first recurrence	41 (10.7)	22 (8.5)	19 (15.3)	0.042
At other time during the follow-up	38 (9.9)	20 (7.7)	18 (14.5)	0.036
Phenotypes of HRBM^b^				–
Tumor size and/or number	40 (50.6)	20 (47.6)	20 (54.1)	0.568
Vascular invasion (VI)	13 (16.5)	7 (16.7)	6 (16.2)	0.957
Metastasis	12 (15.2)	7 (16.7)	5 (13.5)	0.921
Tumor size and/or number and VI	6 (7.6)	3 (7.1)	3 (8.1)	1.000^c^
Tumor size and/or number and metastasis	5 (6.3)	3 (7.1)	2 (5.4)	1.000^c^
VI and metastasis	1 (1.3)	1 (2.4)	0 (0.0)	1.000^c^
Tumor size and/or number, VI and metastasis	2 (2.5)	1 (2.4)	1 (2.7)	1.000^c^
Median TRBM (months)				
Before PSM (95% CI)	–	Not available	101.4 (67.3–135.5)	<0.001

TRBM, time to recurrence beyond the Milan criteria; PSM, propensity score matching; HBV, hepatitis B virus; HRBM, hepatocellular carcinoma recurrence beyond the Milan criteria; VI, vascular invasion.

a Among the HCC patients in the whole cohort or in each group.

b Among the HCC patients with recurrence beyond the Milan criteria in the whole cohort or each group.

c Fisher’s exact test.

**Table 4:** Patterns of HRBM after resection or ablation as first-line therapy for patients with single-nodule HBV-associated HCC of 2–3 cm after PSM.

Patterns of HRBM	Total*n* = 212 (%)	According to treatment		
		Resection (*n* = 106)	Ablation (*n* = 106)	*P*-value
Recurrence beyond the Milan criteria^a^	52 (24.5)	19 (17.9)	33 (31.1)	0.025
At first recurrence	23 (10.8)	6 (5.7)	17 (16.0)	0.015
At other time during the follow-up	29 (13.7)	13 (12.3)	16 (15.1)	0.549
Phenotypes of HRBM^b^				–
Tumor size and/or number	27 (51.9)	9 (47.4)	18 (54.5)	0.618
Vascular invasion (VI)	10 (19.2)	4 (21.1)	6 (18.2)	1.000
Metastasis	6 (11.5)	1 (5.3)	5 (15.2)	0.397^c^
Tumor size and/or number and VI	5 (9.6)	2 (10.5)	3 (9.1)	1.000^c^
Tumor size and/or number and metastasis	2 (3.8)	2 (10.5)	0 (5.4)	0.129^c^
VI and metastasis	1 (1.9)	1 (2.4)	0 (0.0)	0.365^c^
Tumor size and/or number, VI and metastasis	1 (1.9)	0 (0.0)	1 (3.0)	1.000^c^
Median TRBM (months)				
After PSM (95%CI)	–	Not available	85.7 (57.2–114.2)	<0.001

TRBM, time to recurrence beyond the Milan criteria; PSM, propensity score matching; HBV, hepatitis B virus; HRBM, hepatocellular carcinoma recurrence beyond the Milan criteria; VI, vascular invasion.

a Among the HCC patients in the whole cohort or in each group.

b Among the HCC patients with recurrence beyond the Milan criteria in the whole cohort or each group.

c Fisher’s exact test.

**Table 5:** Univariate and multivariate analyses of the occurrence of HRBM in the entire cohort.

Variable	Univariate analysis		Multivariate analysis	
	HR (95% CI)	*P*-value	HR (95% CI)	*P*-value
Gender (female *vs*. male)	0.825 (0.407–1.670)	0.593	–	–
Age (years)	1.020 (0.995–1.046)	0.117	–	–
AFP (ng/ml)	1.000 (1.000–1.000)	0.597	–	–
ALB (g/L)	0.927 (0.871–0.986)	0.015	–	0.613
TBIL (µmol/L)	0.998 (0.980–1.016)	0.841	–	–
CRP (≥1.81 *vs*. <1.81 mg/L)	1.706 (1.029–2.827)	0.038	1.783 (1.052–3.023)	0.032
PLT (≤80 × 10^9^/L *vs*. >80 × 10^9^/L)	4.142 (2.194–7.817)	<0.001	3.579 (1.856–6.905)	<0.001
WBC (×10^9^/L)	0.918 (0.793–1.063)	0.254	–	–
PT (s)	1.169 (0.970–1.410)	0.101	–	–
Size (mm)	0.996 (0.922–1.076)	0.917	–	–
ALBI (grade 2 *vs*. grade 1)	2.441 (1.416–4.208)	0.001	–	0.280
Treatment (ablation *vs*. resection)	2.207 (1.330–3.665)	0.002	1.975 (1.148–3.334)	0.014

Data in parentheses are 95% CIs. The data were analyzed using the binary logistic regression, and the variables with P-value <0.1 in univariate binary logistic regression were entered into the multivariate model. Significance level: 0.05.

AFP, alpha-fetoprotein; ALB, albumin; TBIL, total bilirubin; CRP, C-reactive protein; PLT, platelet; WBC, white blood cell; PT, prothrombin time; ALBI, albumin–bilirubin.

Besides, **Tables 3**, **4** show that whether it was in the entire cohort or in the resection group and the ablation group, these simple recurrence phenotypes were the most common phenotypes of HRBM, among which the phenotype of tumor size and/or number (i.e., at least one within three lesions >3 cm, or one lesion >5 cm, or the number of lesions >3, and no evidence of extrahepatic metastasis and vascular invasion) was the most common and accounted for more than 50% of various phenotypes of HRBM.

### Recurrence-Free Survival and Overall Survival

The univariate and multivariate Cox regression of RFS showed that age, TBIL, PLT, and treatment (ablation *vs*. resection) were independent predictors of RFS (**Table 6**). Besides, the univariate and multivariate Cox regression of OS suggested that age, ALBI grade (grade 2 *vs*. grade 1), and treatment (ablation *vs*. resection) were independent predictors of OS (**Table 7**).

**Table 6:** Univariate and multivariate analyses of recurrence-free survival (RFS) in the entire cohort.

Variable	Univariate analysis		Multivariate analysis	
	HR (95% CI)	*P*-value	HR (95% CI)	*P*-value
Gender (female *vs*. male)	0.850 (0.530–1.361)	0.499	–	–
Age (years)	1.021 (1.004–1.037)	0.014	1.018 (1.002–1.035)	0.030
AFP (ng/ml)	1.000 (1.000–1.000)	0.956	–	–
ALB (g/L)	0.961 (0.922–1.001)	0.054	–	0.811
TBIL (µmol/L)	1.016 (1.008–1.024)	<0.001	1.016 (1.006–1.025)	0.001
CRP (≥1.81 *vs*. <1.81 mg/L)	1.278 (0.919–1.778)	0.144	–	–
PLT (≤80 × 10^9^/L *vs*. >80 × 10^9^/L)	2.107 (1.410–3.148)	<0.001	1.591 (1.048–2.414)	0.029
WBC (×10^9^/L)	0.992 (0.903–1.091)	0.875	–	–
PT (s)	1.075 (0.988–1.170)	0.094	–	0.786
Size (mm)	0.986 (0.938–1.038)	0.593	–	–
ALBI (grade 2 *vs*. grade 1)	1.724 (1.206–2.464)	0.003	–	0.543
Treatment (ablation *vs*. resection)	2.059 (1.481–2.862)	<0.001	1.794 (1.275–2.525)	0.001

Data in parentheses are 95% CIs. The data were analyzed using the Cox regression, and the variables with P value <0.1 in univariate Cox regression were entered into the multivariate model. Significance level: 0.05.

AFP, alpha-fetoprotein; ALB, albumin; TBIL, total bilirubin; CRP, C-reactive protein; PLT, platelet; WBC, white blood cell; PT, prothrombin time; ALBI, albumin–bilirubin.

**Table 7:** Univariate and multivariate analyses of overall survival (OS) in the entire cohort.

Variable	Univariate analysis		Multivariate analysis	
	HR (95% CI)	*P*-value	HR (95% CI)	*P*-value
Gender (female *vs*. male)	1.233 (0.470–3.234)	0.670	–	–
Age (years)	1.058 (1.017–1.102)	0.006	1.042 (1.000–1.086)	0.051
AFP (ng/ml)	1.000 (1.000–1.000)	0.402	–	–
ALB (g/L)	0.859 (0.796–0.927)	<0.001	–	0.716
TBIL (µmol/L)	1.019 (0.974–1.067)	0.407	–	–
CRP (≥1.81 *vs*. <1.81 mg/L)	0.985 (0.458–2.119)	0.969	–	–
PLT (≤80 × 10^9^/L *vs*. >80 × 10^9^/L)	3.452 (1.571–7.583)	<0.001	–	0.299
WBC (×10^9^/L)	0.744 (0.580–0.955)	0.020	–	0.230
PT (s)	1.166 (1.032–1.319)	0.014	–	0.973
Size (mm)	1.010 (0.900–1.134)	0.864	–	–
ALBI (grade 2 *vs*. grade 1)	5.141 (2.472–10.691)	<0.001	3.652 (1.728–7.718)	0.001
Treatment (ablation *vs*. resection)	5.056 (2.347–10.894)	<0.001	4.226 (1.938–9.215)	<0.001

Data in parentheses are 95% CIs. The data were analyzed using the Cox regression, and the variables with P-value <0.1 in univariate Cox regression were entered into the multivariate model. Significance level: 0.05. The multivariate analysis was selected by the approach of stepwise forward (LR).

AFP, alpha-fetoprotein; ALB, albumin; TBIL, total bilirubin; CRP, C-reactive protein; PLT, platelet; WBC, white blood cell; PT, prothrombin time; ALBI, albumin–bilirubin.

**Figure 3:**
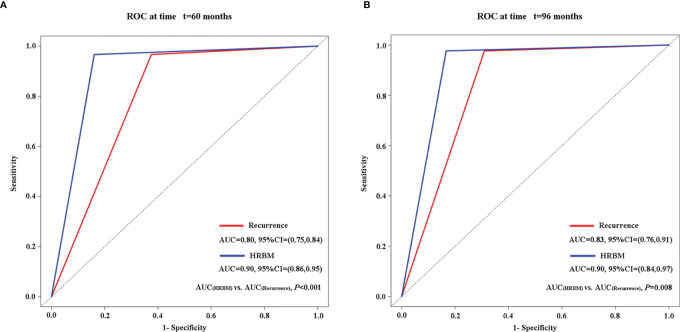
Comparison of the ability of HRBM and recurrence to predict overall survival by the time-dependent ROC and the estimated AUC at 5 years **(A)** and 8 years **(B)**. AUC, area under the curve; HRBM, HCC recurrence beyond the Milan criteria; ROC, receiver operating characteristic.

In addition, we performed time-dependent ROC curves and calculated the estimated AUC to compare the ability of the HRBM and recurrence in predicting OS at 5 and 8 years, which suggested that compared with recurrence, the HRBM has more obvious advantages in the prediction of OS in patients with single-nodule HBV-associated HCC of 2–3 cm after curative treatment at 5 (AUC_Recurrence_ = 0.80, 95% CI: 0.75–0.84; AUC_HRBM_ = 0.90, 95% CI: 0.86–0.95; AUC_HRBM_ *vs*. AUC_Recurrence_, *P* < 0.001) (shown in **Figure 3A**) and 8 (AUC_Recurrence_ = 0.83, 95% CI: 0.76–0.91; AUC_HRBM_ = 0.90, 95% CI: 0.84–0.97; AUC_HRBM_ *vs*. AUC_Recurrence_, *P* = 0.008) years (shown in **Figure 3B**).

## Discussion

In this comparative study, we found that the incidence of HRBM in the ablation group was significantly higher than that in the resection group in patients with single-nodule HBV-associated HCC of 2–3 cm. Moreover, our study also showed that compared with ablation, resection provided longer median TRBM for those patients. The independent risk factors for TRBM after curative treatment included older age and serum CRP ≥1.81 mg/L and PLT ≤80 × 10^9^/L and treatment (ablation).

Ablation and resection were regarded as the first-line treatment for patients with early HCC <2 cm by most institutions in more cases because of the scarcity of donors used for liver transplantation, transplant rejection, and the long-term usage of immunosuppressive preparation after transplantation (20). However, for patients with early HCC of 2–3 cm, whether ablation can be used as a first-line option deserves further consideration compared with resection (4–11). In our opinion, HRBM should be responsible considerably for this controversy, because of its significant relations to the prognosis of patients who received curative ablation (12, 13). For example, Adam et al. reported retrospectively that 66.1% of potentially transplantable patients with solitary HCC ≤3 cm who underwent RFA as first-line therapy had recurrence, of which 41.7% were the HRBM, and this study suggested that the difference in tumor size (HCC ≤ 2 cm *vs*. HCC > 2 cm) led to the significant difference in the incidence of HRBM in those patients after curative RFA and further led to significantly different overall survival (12). Our study showed that compared with recurrence, the HRBM was a stronger predictor of overall survival in patients with single-nodule HBV-associated HCC of 2–3 cm and also suggested that curative treatment (ablation *vs*. resection) led to the significant difference in the incidence of HRBM in those patients and further led to significantly different overall survival. In addition to treatment (ablation), we also identified other independent risk factors (i.e., serum CRP ≥ 1.81 mg/L and PLT ≤ 80 × 10^9^/L) for the occurrence of HRBM after curative treatment by the binary logistic regression. However, the baseline data of previously published studies on resection versus ablation in patients with HCC of 2–3 cm did not include both CRP and PLT (4–11), which may cause significantly different incidences of HRBM in those patients undergoing surgery and ablation and further come to different conclusions.

Besides, the comparison of the differences in the patterns of HRBM after curative resection and ablation can provide an important reference for those transplantable candidates to choose a more appropriate first-line treatment before transplantation, and its significant reason was that the prognosis of HCC patients with recurrence within the Milan criteria who received salvage liver transplantation is significantly better than those who received re-ablation or re-resection (14). In this study, we found that resection was more suitable as a first-line treatment for patients with single-nodule HBV-associated HCC of 2–3 cm compared with ablation, because patients who underwent resection as first-line treatment can obtain the longer median TRBM and lower incidence of HRBM than patients who received ablation as first-line treatment, so that they can get more opportunities for salvage transplantation and buy more waiting time for salvage transplantation.

In addition to treatment (ablation), the multivariate Cox regression and the binary logistic regression also showed that serum CRP ≥1.81 mg/l and PLT ≤80 × 10^9^/L were independent risk factors of TRBM and the occurrence of HRBM. Regarding C-reactive protein (CRP), a study showed that elevated CRP, an acute phase protein, was correlated closely with portal vein tumor thrombus and distant metastasis in patients with HCC (21). Besides, more and more studies showed that increased serum CRP was a reliable and cost-effective biomarker for the shorter recurrence-free survival and overall survival in patients with HCC (22–25). PLT was regarded as an important participant in hepatitis-related chronic liver damage by hepatic stellate cell (HSC)-related inflammatory processes, which caused thrombocytopenia (26). Besides, thrombocytopenia was considered a strong associated biomarker with the development of hepatitis-associated HCC (27). Furthermore, thrombocytopenia was an effective and inexpensive indicator of predicting HCC recurrence (28). Our previous study also showed that thrombocytopenia was significantly related to shorter median overall survival (OS) (18).

Regarding the patterns of HRBM, we found that the phenotype of tumor size and/or number (i.e., at least one within three lesions >3 cm, or one lesion >5 cm, or the number of lesions >3, and no evidence of extrahepatic metastasis and vascular invasion) was the most common pattern and accounted for more than 50% whether after radical resection or ablation or not. Therefore, it was very important to effectively treat HCC with HRBM as early as possible to prevent the progression and even downstage it back to HCC within the Milan criteria (29).

Although our research is so meaningful, there are some limitations in our study. Firstly, this study has a single-centered and retrospective study design; therefore, selection bias is easily formed. However, propensity score matching analysis, which can reduce selection bias to some degree, further verified our conclusion. Secondly, the follow-up time of this study was relatively short, especially in the ablation group, so that the HRBM in those patients occurs incompletely; therefore, the calculation of the patterns of HCC HRBM in patients with single-nodule HBV-associated HCC of 2–3 cm after curative resection and ablation may not be accurate enough.

In conclusion, our study revealed that compared with ablation, resection should be considered as a more appropriate first-line option for patients with single-nodule HBV-associated HCC of 2–3 cm because of its significantly lower incidence of HRBM related with significantly longer overall survival, and resection is a more promising bridging treatment for liver transplantation in those transplantable candidates because of the longer median TRBM and its lower incidence of HRBM.

## Data Availability Statement

The raw data supporting the conclusions of this article will be made available by the authors, without undue reservation.

## Ethics Statement

The studies involving human participants were reviewed and approved by Sun Yat-sen University Cancer Center Hospital Ethics Committee. Written informed consent was not provided because of the retrospective nature of this study.

## Author Contributions

This study was conceived and designed by SC and WF. Study material and access to the patients were provided by WF. Data were acquired by SC, WM, YW, LS, HQ, FC, TH, and WF. Data were analyzed by SC, WM, YW, LS, HQ, FC, TH, and WF. The manuscript was drafted by SC, WM, YW, LS, HQ, FC, TH, and WF. All authors contributed to the draft and critically reviewed or revised the manuscript.

## Funding

This study was supported by the National Natural Science Foundation of China (No. 81771954) and Guangdong Province Key Field Research and Development Project (2019B110233001).

## Conflict of Interest

The authors declare that the research was conducted in the absence of any commercial or financial relationships that could be construed as a potential conflict of interest.

## Publisher’s Note

All claims expressed in this article are solely those of the authors and do not necessarily represent those of their affiliated organizations, or those of the publisher, the editors and the reviewers. Any product that may be evaluated in this article, or claim that may be made by its manufacturer, is not guaranteed or endorsed by the publisher.

## Abbreviations

ALB, albumin; ALBI, albumin–bilirubin; AFP, alpha-fetoprotein; AUC, estimated area under the curve; CRP, C-reactive protein; CT, computed tomography; CI, confidence interval; HBV, hepatitis B virus; HRBM, hepatocellular carcinoma recurrence beyond the Milan criteria; HCC, hepatocellular carcinoma; HR, hazard ratio; IQR, interquartile range; MRI, magnetic resonance imaging; OS, overall survival; PLT, platelet; PT, prothrombin time; PSM, propensity score matching; RFS, recurrence-free survival; ROC, time-dependent receiver operating characteristic; TRBM, time to recurrence beyond the Milan criteria; TBIL, total bilirubin; US, ultrasound; VI, vascular invasion; WBC, white blood cell.
